# Human body weight, nutrients, and foods: a scoping review

**DOI:** 10.29219/fnr.v66.8814

**Published:** 2022-08-22

**Authors:** Jøran Hjelmesæth, Agneta Sjöberg

**Affiliations:** 1 Morbid Obesity Centre, Department of Medicine, Vestfold Hospital Trust, Tønsberg, Norway;; 2 Department of Endocrinology, Morbid Obesity and Preventive Medicine, Institute of Clinical Medicine, University of Oslo, Norway;; 3 Department of Food and Nutrition, and Sport Science, University of Gothenburg, Gothenburg, Sweden

**Keywords:** Scoping review, Body weight, Human, Macronutrients, Foods, Overweight, Obesity, Diet, Adults, Children

## Abstract

**Background:**

The aim of this article (scoping review) is to elucidate the current knowledge for the potential role of body weight for setting and updating Dietary Reference Values (DRVs) and Food-Based Dietary Guidelines (FBDGs). The following research questions were formulated:

1. What is known about the association between intakes of specific nutrient and/or foods (exposure/intervention) and body weight (outcome) in the general population?
2. What is known about the association between body weight (exposure) and intakes of specific nutrient and/or foods (outcomes)?
3. Is there any evidence suggesting specific effects of foods or nutrients on body weight independent of caloric content?

**Methods:**

To identify potentially relevant articles, PubMed was searched from January 1, 2011 to June 9, 2021. The search strategy was drafted by the NNR2022 Committee. The final results were exported into EndNote. Systematic reviews (SRs), scoping reviews (ScRs), reviews, and meta-analyses (MAs) on the topic ‘Body weight’ published between January 1, 2011 and June 9, 2021, including human participants from the general population, in English or Scandinavian language (Norwegian, Swedish, or Danish), were considered eligible.

**Main findings:**

First, the overall body of evidence based on findings from SRs and MAs of observational and clinical studies indicates that changes in intakes of specific nutrients (sugar, fiber, and fat) and/or foods (sugar sweetened beverages, fiber rich food, and vegetables) are associated with modest or small short-term changes (0.3–1.3 kg) in body weight in the general population (with or without obesity/overweight), while long-term studies are generally lacking. Second, no study in our search assessed any association between body weight (exposure) and intakes of specific nutrients or foods (outcomes). Third, limited evidence suggests, but does not prove, that some foods or nutrients may have specific effects on body weight or body weight measures independent of caloric content (e.g. nuts and dairy). These findings may inform the setting and updating of DRVs and FBDGs in NNR2022.

## Popular scientific summary

- Higher intakes of sugar, sugar sweetened beverages, and saturated fat are associated with small short-term increases in bodyweight (≤1 kg).
- Higher intakes of fiber, fiber-rich food, and vegetables are associated with small short-term decreases in bodyweight (≤1 kg).
- Longer-term (>1 year) studies are generally lacking.
- No study assessed whether higher bodyweight may cause increased food intake.
- Some foods may affect body weight independent of caloric content.

## Rationale

Obesity is a chronic disease, which is associated with increased risk for several Noncommunicable diseases (NCDs), including cardiovascular diseases, type 2 diabetes, some cancers, and chronic respiratory diseases, including obstructive sleep apnea (WHO/Europe|WHO European Regional Obesity Report 2022). In 2016, the age standardized prevalence of adult overweight (including obesity) in the Nordic-Baltic region varied between 55% (Denmark) and 60% (Lithuania), with an obesity prevalence between 20% (Denmark) and 26% (Lithuania). Using the WHO growth reference, the prevalence of overweight (including obesity) among school-aged children varied from 23% (Estonia) to 31% (Iceland), and among adolescents from 19% (Lithuania) to 27% (Iceland). Despite several action plans to stop the obesity epidemic, the prevalence of overweight and obesity in the WHO European Region has increased, and no member state seems to reach the target of halting the rise in obesity by 2025 (WHO/Europe|WHO European Regional Obesity Report 2022).

It is well known that body weight (outcome) is influenced by the balance between energy intake and energy expenditure, which both may be affected by dietary patterns and food and nutrient intakes (exposures) (1). However, the opposite is also true, as body composition influences both energy expenditure (resting metabolic rate) and intake (2). Therefore, people with obesity have to struggle against both increased appetite and diminished satiation and satiety before, during, and after meals (2). Furthermore, there is an ongoing debate whether the simple energy balance theory is sufficient to explain differences in body weight, and particularly, whether some foods or nutrients affect body weight beyond their intrinsic caloric content (3). However, energy balance will be more specifically addressed in another chapter of NNR2022.

The NNR2012 (Nordic Nutrition Recommendations 2012 Integrating nutrition and physical activity ISBN 978-92-893-2670-4, link Nordic Nutrition Recommendations 2012|Nordic cooperation (norden.org)) concluded as follows about the relationship among dietary patterns, nutrients, and overweight/obesity (pp. 36–37).

First, ‘the evidence linking a higher dietary fibre intake to reduced weight gain is clear’ (prospective studies). Second, ‘No other evident associations between macronutrient and weight change in adults were observed in NNR systematic review (SR) on diet and long term weight change’. ‘However, combined results from intervention studies not designed for intentional weight loss show that reduced total fat intake was associated with a modest weight reduction’. Third, ‘Also, reduced intake of sugar and sugar sweetened beverages (SSBs) has been associated with modest weight loss’. Fourth, ‘there is clear evidence that fibre-rich foods (examples) and perhaps also dairy products, are associated with reduced weight gain’. Fifth, ‘refined cereals, sugar-rich foods and drinks, red meat, and processed meat are associated with increased weight gain in long-term studies’.

This scoping review (ScR) aims to assess newer evidence published after the SR implemented in NNR2012 to inform other chapters in NNR2022.

### Objectives

This ScR of reviews was conducted in order to ‘systematically map the evidence or any gaps in knowledge of the associations between intakes of specific nutrient and/or foods and body weight’. The aim of this chapter is to elucidate the current knowledge for the potential role of body weight for setting and updating DRVs and FBDGs.

The following research questions were formulated:

1. What is known about the association between intakes of specific nutrient and/or foods (exposure/intervention) and body weight (outcome) in the general population?
2. What is known about the association between body weight (exposure) and intakes of specific nutrient and/or foods (outcome)?
3. Is there any evidence suggesting specific effects of foods or nutrients on body weight independent of caloric content?

## Methods

### Protocol and registration

This ScR follows the protocol developed within the NNR2022 project (‘The Nordic Nutrition Recommendations 2022 – Instructions to authors of chapter’), and the protocol can be found on the official NNR2022 website (Please, see link https://www.helsedirektoratet.no/english/nordic-nutrition-recommendations-2022). The sources of evidence used in the chapter follow the eligibility criteria described in the paper ‘The Nordic Nutrition Recommendations 2022 – Principles and methodologies’ published in *Food & Nutrition Research* (4). We have followed the procedures of the PRISMA Extension for Scoping Reviews (PRISMA-ScR) defined by the EQUATOR Network (5). None *de novo* NNR2022 SRs or qualified SRs are included as source of evidence in the chapter ‘The Nordic Nutrition Recommendations 2022 – prioritisation of topics for de novo systematic reviews’ (6).

### Eligibility criteria

Systematic reviews, ScRs, reviews, and meta-analyses (MAs) on the topic ‘Body weight’ published between January 1, 2011 and June 9, 2021, including human participants from the general population, in English or Scandinavian language (Norwegian, Swedish, or Danish), were considered eligible.

### Exclusion criteria

Articles with incorrect language, population, intervention/exposure, outcome, or study design were excluded from further review:

1. Language (non-English or non-Scandinavian)
2. Population clearly different from the general population or people with disease (e.g. cancer, cardiovascular disease, diabetes, hypertension, and psychiatric disease)
3. Interventions or exposures not directly related to food or nutrient intake (i.e. exercise, drugs, surgery, supplements, genetics, epigenetics, non-specific multicomponent intervention including behavioral therapy and/or exercise, other or no defined intervention/exposure, and physiological mechanisms)
4. Outcomes other than body weight or intakes of food/nutrients (e.g. obesity-related diseases and other)
5. Study design (not review, ScR, SR, or meta-analysis)

### Information sources

To identify potentially relevant articles, PubMed was searched from January 1, 2011 to June 9, 2021. The search strategy was drafted by the NNR2022 Committee. The final results were exported into EndNote.

### Search

PubMed was searched with the search terms ‘Body AND weight’ restricted to

1. Body and weight
2. Publication date from January 1, 2011 to June 9, 2021
3. Filter: Humans
4. Publication type: Review/SR/meta-analysis
5. Topic title

Body[TI] AND weight[TI] – Search Results – PubMed (nih.gov)

### Selection of sources of evidence

Both reviewers screened the same 443 publications (titles and abstracts), discussed the results, and amended the screening and data extraction manual before beginning screening for this review. Both sequentially evaluated the titles, abstracts, and then full text of all publications identified by our searches for potentially relevant publications. We resolved disagreements on study selection and data extraction by consensus.

### Data charting process

A data-charting form was developed to determine which variables to extract. The two reviewers independently charted the data, discussed the results, and continuously updated the data charting form in an iterative process.

### Data items

We abstracted (categorized) data on year of publication, study design, country of origin, aim, population, intervention/exposure/outcomes, and key findings.

### Synthesis of Results

We grouped the publications by the major exposures/interventions such as foods and/or macronutrients and non-nutritive sweeteners (NNS) to meet the objectives of the ScR. The evidence is presented in a narrative format in text and table and grouped as A) sugar intake, SSBs, and non-caloric/low-calorie sweeteners (NNSs); B) whole grain and fiber; C) vegetables, fruits, nuts, and seeds; D) dairy/dairy proteins; E) diets according to food or macronutrient composition; F) macronutrient composition – fats; G) water, tea, coffee, and alcohol; and H) meat.

## Results

### Selection of sources of evidence

**Fig. 1:**
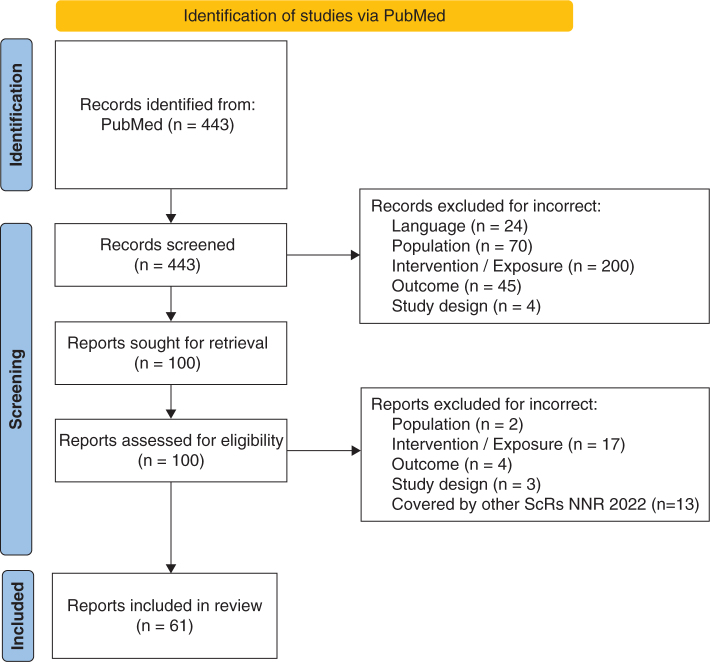
Flow diagram. From: Page MJ, McKenzie JE, Bossuyt PM, Boutron I, Hoffmann TC, Mulrow CD, et al. The PRISMA 2020 statement: an updated guideline for reporting systematic reviews. BMJ 2021;372:n71. doi: 10.1136/bmj.n71

The search revealed 443 citations from which the titles and abstracts were initially assessed for eligibility. A total of 343 citations were excluded for the following reasons: incorrect language (*n* = 24), incorrect population (*n* = 70), incorrect intervention/exposure (*n* = 200), incorrect outcome (*n* = 45), or incorrect study design (*n* = 4) (Fig. 1, flow diagram). The remaining 100 citations were evaluated as full text articles, and additionally, 26 citations were excluded due to incorrect population (*n* = 2), incorrect intervention/exposure (*n* = 17), incorrect outcome (*n* = 4), or incorrect study design (*n* = 3), resulting in 74 publications. In addition, 13 citations were excluded because they were covered by other ScRs in NNR2022 (meal frequency/intermittent dieting/time restricted feeding (*n* = 9) and energy (*n* = 4)), leaving 61 citations eligible for the ScR.

### Characteristics of sources of evidence

The results according to relevant intervention or exposure categories in the 61 publications are presented in Table 1. The number of studies available per intervention/exposure varied from 1 (meat) to 13 (sugar intake, SSBs, and NNSs). None of the publications assessed the effect of overweight/obesity on food/nutrient intake.

**Table 1:** Results according to intervention/exposure category

First author, year publication	Ref.	Study design, number included in analysis	Country	Aim	Population	Intervention, exposure, and outcomes	Key findings
A) Sugar intake or SSBs (SSB, *n* = 7) and non-nutritive sweeteners (NNS, *n* = 6)							
Bes-Rastrollo M, 2016	(7)	Narrative review of systematic reviews (SR) and meta-analyses (MAs) (*n* = 23)	Spain	‘To summarize the evidence from the SRs and MAs we conducted a comprehensive literature review to include those SRs based on the topic of SSBs as a potential risk factor for weight gain or obesity’.	Human studies	SSB consumption and weight gain or obesity	‘Added sugars, especially SSB consumption, are an important risk factor for weight gain and obesity’. ‘It seems that the effect of fructose on weight gain is mediated through an overconsumption of beverages with fructose-containing caloric sweeteners leading to an extra provision of energy intake’.
Kahn R, 2014	(8)	Point narrative review	USA	To discuss whether ‘any dietary or added sugar has a unique or detrimental impact relative to any other source of calories on the development of obesity or diabetes’.	Not addressed	Sugar and obesity/diabetes	‘If there are any adverse effects of sugar, they are due entirely to the calories it provides, and it is therefore indistinguishable from any other caloric food. Excess total energy consumption seems far more likely to be the cause of obesity and diabetes’.
Bray GA, 2014	(9)	Point narrative review	USA	‘We provide our opinion and review of the data to date that we need to reconsider consumption of dietary sugar based on the growing concern of obesity and type 2 diabetes’.	Not addressed	Sugar and obesity/diabetes	‘Consumption of calorie-sweetened beverages has continued to increase and plays a role in the epidemic of obesity, the metabolic syndrome, and fatty liver disease. Reducing intake of soft drinks is associated with less weight gain’.
Massougbodji J, 2014	(10)	SR of reviews (*n* = 20) (2006–2013) eight SR and 12 non-SR	USA	Objectives: ‘1) identify published reviews on the relation between SSBs and body weight, 2) assess the scientific quality of these reviews by using two different scoring systems, 3) position the authors’ conclusions on a Likert scale ranging from 0 = no evidence of a causal relation to 5 = strong evidence of a causal relation, and 4) identify study characteristics associated with the authors’ position, including the quality scores and source of funding’.	Adults, children, and adolescents	SSBs and body weight	‘In conclusion, many reviews that have examined the association between SSB consumption and obesity/weight gain were recently published, but there is no consensus on the strength of the evidence on causality. As measured by two quality-assessment tools, the methodologic quality of the reviews did not explain the orientation of the authors’ conclusions’. ‘We found that reviews funded by the industry were less likely to conclude that there was a strong association between SSB consumption and obesity/weight gain’.
Ebbeling CB, 2014	(11)	Narrative review	USA	‘The purpose of this review is not to underscore the debate but rather to consider how recently published data pertaining to SSBs contribute to the evidence base for preventing and treating obesity, with application to caring for patients’.	Adults and children	SSBs and body weight/obesity	‘Available data provide an evidence base for counselling patients to reduce consumption of SSBs’.
Sievenpiper JL, 2012	(12)	SR of intervent studies and MA (*n* = 41). 18 (58%) isocaloric and 5 hypercaloric (50%) trials were randomized.	Canada	‘To review the effects of fructose on body weight in controlled feeding trials’.	Adults	Fructose and body weight	‘Our aggregate analyses of the effects of fructose in 31 trials with isocaloric comparisons (637 participants) and 10 trials with hypercaloric comparisons (119 participants) showed divergent results. The isocaloric trials did not provide consistent evidence for a body weight–increasing effect of fructose, whereas the hypercaloric trials did’.
Te Morenga L, 2012	(13)	SR and MA of RCTs (*n* = 19 ad libitum, 11 isoenergetic), duration 2-week to 6-month, and CHSs (*n* = 38), minimum duration 1-year	New Zealand	‘To summarise evidence on the association between intake of dietary sugars and body weight in adults and children’.	Adults and children	Dietary sugars and body weight Ad libitum diets, variation in sugar intakes, and isoenergetic exchange of sugars with other carbohydrates Intake of sugar sweetened beverages with 1-year follow-up comparing lowest to highest intake in relation to intakes of sugar sweetened beverages after 1-year follow-up in prospective studies.	Reduced intake of dietary sugars showed a decrease in body weight (0.80 kg, 95% CI 0.39 to 1.21); increased intake of sugars showed a weight increase (0.75 kg, 0.30 to 1.19). Isoenergetic replacement of dietary sugars with other carbohydrates resulted in no change in body weight (0.04 kg, −0.04 to 0.13). ‘The odds ratio for being overweight or obese was 1.55 (1.32 to 1.82) among groups with the highest intake compared with groups with the lowest intake’. The change in body fatness after modification of sugar intake seemed to be mediated by changes in energy intake.
Mattes RD, 2011	(14)	SR and MA (*n* = 10) of randomized experiments	USA	To critically review data from RCTs and evidence-based reviews through January 2009 concerning effects of consumption of nutritively sweetened beverages (NSBs) on changes in body weight and adiposity.	Children, adolescents, and adults	Nutritively sweetened beverages and body weight	‘Meta-analysis of studies providing access to results separately for subjects overweight at baseline showed a significant effect of a roughly 0.35 standard deviations lesser BMI change (i.e. more weight loss or less weight gain) relative to controls. The current evidence does not demonstrate conclusively that NSB consumption has uniquely contributed to obesity or that reducing NSB consumption will reduce BMI levels in general’.
Laviada-Molina, 2020	(15)	SR and MA of RCTs, *n* = 20, 2,914 particip. 4–77 weeks 15/20 studies shorter than 48 weeks.	Mexico/UK	‘To assess the effects of nonnutritive sweeteners (NNS) on body weight’	General population, both subjects with healthy weight and subjects with overweight/obesity of all ages, no conditions or comorbidities	NNS vs sucrose/placebo/water/nothing. Primary outcome: difference in body weight between NNS and comparators. NNS, also known as non-caloric/very low-calorie sweeteners or artificial sweeteners such as aspartame, saccharin, sucralose, stevia, cyclamate, and acesulfame-K.	‘Participants consuming NNS showed significant weight/BMI differences favouring NNS compared with nonusers (1.3 kg)’. ‘NNS versus placebo/no intervention and NNS versus water produced no effect’. ‘When comparing NNS versus sucrose, significant weight/BMI differences appeared favouring NNS’. ‘Data suggest that replacing sugar with NNS leads to weight reduction, particularly in participants with overweight/obesity under an unrestricted diet’.
Cavagnari BM, 2019	(16)	Special article-narrative review of studies with several study designs	Argentina	‘Main objective of this article is to review the available evidence on the consumption of non-caloric sweeteners in relation to body weight’.	Children and adults	Non-caloric sweeteners and body weight	‘Currently, the higher quality of evidence (RCTs, SRs and meta-analyzes of RCTs) shows that consumption of NCS -in replacement of sugars- could be useful for reducing calorie intake and relative body weight in adults’.
Mosdøl A, 2018	(17)	Scoping review of reviews (*n* = 40, *n* = 5 SRs) with several study designs	Norway	‘To determine the extent and type of summarized evidence published the last 10 years regarding the potential effects of intense sweeteners on appetite and weight change’.	Children and adults	Intense sweeteners Weight change	‘Apart from the observational studies, the presented primary evidence in humans is dominated by small studies with short follow-up considered insufficient to assess weight change’. ‘With few exceptions, the reviews on intense sweeteners and weight change underuse systematic methodology, and thus, the available evidence’.
Rogers PJ, 2016	(18)	SR and MA of RCTs and prospective cohort studies (*n* = 79), mainly short term (<1 day), nine RCT intervention studies >1 day	UK	‘To bring together the totality of evidence to test the primary (null) hypotheses that low-energy sweeteners (LES) consumption per se or as a replacement for caloric sweeteners in foods or beverages has no effect on EI or BW outcomes in adults or children’.	Children and adults and animals	Low-energy sweeteners (LES) versus sugar or water and energy intake and body weight	‘We found a considerable weight of evidence in favour of consumption of LES in place of sugar as helpful in reducing relative EI and BW, with no evidence from the many acute and sustained intervention studies in humans that LES increase EI. Importantly, the effects of LES-sweetened beverages on BW also appear neutral relative to water, or even beneficial in some contexts’.
Miller PE, 2014	(19)	SR and MA of RCTs (*n* = 15) and prospective cohort studies (PCS) (*n* = 9)	USA	‘To systematically review and quantitatively evaluate results from RCTs and prospective cohort studies, separately, that examined the relation between low-calorie sweeteners and body weight, fat mass, BMI, and waist circumference’.	Adults and children	Low-calorie sweeteners and body weight	Substituting Low-calorie sweeteners (LCS) for sugar modestly reduced body weight (0.80 kg), BMI, fat mass, and waist circumference. Among prospective cohort studies, LCS intake was not associated with body weight or fat mass, but was significantly associated with slightly higher BMI (0.03; 95% CI: 0.01, 0.06).
B) Whole grain (WG, *n* = 5) and fiber (*n* = 3)							
Maki KC, 2019	(20)	Review and MA of RCTs (*n* = 9, *n* of patients = 973, 12- to 16-week intervention, and non-RCTs (*n* = 11), and qualitative analysis of prospective cohorts (*n* = 6)	USA	‘To provide an updated quantitative analysis of data from both observational studies and RCTs examining the relationship of whole grain (WG) intake with body weight’	Adults	Primary outcome RCT data meta-analysis change in body weight (kg), diff. between the exposed group with the highest WG intake reported and the control group. WG intake 32 g/d to 215 g/d for the WG interventions and 0–19 g/d for the studies reporting WG daily intake for controls.	‘Higher WG intake is significantly inversely associated with BMI in observational studies but not in RCTs up to 16 weeks in length’. Meta-regression analysis of cross-sectional evidence showed a significant inverse relationship between the WG intake and BMI. ‘Prospective cohort studies support this relationship, with baseline WG intake and change in WG intake generally showing inverse associations with weight change during follow-up periods of four to 20 years, particularly in the studies with larger numbers of subjects’.
Pol K, 2013	(21)	MA of RCTs (*n* = 26) 1988–2012 *N* = 2,060	Denmark	‘To evaluate the evidence from randomized controlled studies for a role of whole grain in terms of body weight and body composition compared with a non-whole-grain or refined-grain control in apparently healthy adults’.	Adults	Whole grain in terms of body weight and body composition compared with a non-whole-grain or refined-grain control	‘The current meta-analysis does not lend credence to a role for whole grain in body weight management. However, we did show that whole grain beneficially affected the percentage of body fat (–0.5%). Studies were of 2–16-week duration, and most studies lasted only 4–6 weeks’. ‘The relatively short duration of intervention studies (16 week or less) may explain the lack of difference in body weight and fat’.
Karl JP, 2012	(22)	Narrative review	USA	To ‘examine the evidence for a role of whole grain (WG) in body weight regulation’.	Adults (mainly) and children	WG and body weight regulation	‘In summary, intervention trials conducted to date have failed to demonstrate beneficial effects of WG intake on body weight regulation despite observational studies consistently demonstrating that high intakes of WG are associated with lower BMI, and the existence of a variety of mechanisms that could result in WG-mediated effects on body weight’.
Bautista-Castaño I, 2012	(23)	SR: cross-sectional (*n* = 22), prospective cohort studies (*n* = 11), and intervention studies (*n* = 5)	Spain	‘To analyze the epidemiological evidence regarding the influence of dietary patterns that include refined and whole-grain bread consumption on an individual’s ponderal status’.	Adults and children	Effect of whole-grain versus refined bread consumption on body weight measures	‘The greater proportion of studies in this review indicated that the groups of food items, dietary patterns, or dietary models that included bread did not adversely affect ponderal status, and that those that included wholegrain bread even provided benefit. In addition, the dietary patterns that included refined bread achieved poorer results than those that contained whole-grain bread, and were associated with a more favorable distribution of abdominal body fat. Of the five studies that related food patterns that included bread to the distribution of abdominal fat, four were associated with refined bread’.
Giacco R, 2011	(24)	Viewpoint/narrative review	Italy	‘To 1) review the available scientific literature on the relation-ship between whole grain consumption and body weight regulation; 2) evaluate the potential mechanisms by which whole grain intake may help reduce overweight and 3) try to understand why results of cross-sectional and prospective epidemiological studies and of clinical trials have produced diverging evidence on this topic’.	Adults (mainly) and adolescents	The relationship between whole grain consumption and body weight regulation	‘All the studies reviewed in this manuscript demonstrate that a higher intake of whole grains is associated with lower BMI in epidemiological studies. However, so far, the results from a few intervention trials investigating whether a whole grain-low calorie diet is able to reduce body weight, have failed to demonstrate a cause/effect relation’.
Jovanovski E, 2020	(25)	SR and MA of RCTs, *n* = 62, 3,877 subjects Duration 4–52 weeks, median 10 weeks	Canada	‘To summarize and quantify the effects of addition of viscous fiber to the diet [agar, alginate, β-glucan, guar gum, konjac, viscous fiber blend (VFB) (konjac, alginate, and xanthan), psyllium, or xanthan gum] compared with an appropriate control (i.e. fiber-free supplement, nonviscous fiber, placebo, background diet) on ≥1 anthropometric measure’	Overweight or normal weight people with/without diabetes or increased (CVD) risk, median (range) age 51 (16–70), BMI 27 (19–33)	Effect of viscous fiber supplemented to an ad libitum diet along with comparator diets. Both whole food sources (i.e. oats and barley) and isolated fibers were included. Median dose of 8 g viscous fiber/d (range: 0.8–36 g/d)	‘Pooled analysis of 62 trials (*n* = 3877) showed reductions in body weight (−0.33 kg), BMI (−0.28), and waist circumference (−0.63 cm), at a median dose of 6.7 g viscous fiber/d (range: 0.8–36 g/d) for a median duration of 10 week (range: 4–52 week). The change in body fat approached significance at −0.78% (95% CI: −1.56%, 0.00%; *P* = 0.05). Reduction in the primary outcome of body weight was significant mainly owing to the effect in overweight/obese individuals (MD: −0.46 kg; 95% CI: −0.75, −0.18 kg) and those with diabetes and metabolic syndrome (MD: −0.45 kg; 95% CI: −0.87, −0.03 kg), populations that would likely benefit the most from the intervention. The magnitude of effect, as indicated in this study, is modest and lacks clinical significance on an individual level’.
Wanders AJ, 2011	(26)	SR of RCTs (for body weight, 59 papers with 66 comparisons)	The Netherlands	‘To summarize the available literature on the relationship between specific dietary fibre types and three outcome variables: subjective appetite, energy intake and body weight’.	Adults	Effects of dietary fiber types and three outcome variables: subjective appetite, energy intake, and body weight.	‘Out of 66 fibre–control comparisons, 39 showed an absolute reduction in body weight (59%). Irrespective of the fibre group, fibre reduced body weight with 1.3% over the complete study period (on average 0.72 kg), which corresponds to a reduction of 0.4% per 4 weeks. All comparisons on dextrins (100%) and marine polysaccharides (100%) reduced body weight. Other fibres with a high effect rate on weight loss were chitosan (86%), fructans (67%) and arabinoxylans (67%). When fibres were grouped according to physicochemical properties, differences between classes were found for more viscous versus less viscous fibres (53% vs.71%), more soluble versus less soluble fibres (53% vs. 74%) and more fermentable versus less fermentable fibres (56% vs. 63%)’.
Rahmani J, 2019	(27)	SR and MA of RCTs (*n* = 20)	Iran	‘To summarize the effect of cereal beta-glucan consumption on body weight, BMI, waist circumference and a total energy intake’.	Adults	Effects of cereal beta-glucan on energy intake, body weight, BMI, or waist circumference. Mean intervention duration of studies: 7.40 ± 7.25 weeks. Dose of betaglucan varied between 0.88 and 9.9 mg/d.	‘Ingestion of beta-glucan containing products improves weight loss and reduces BMI in the intervention group compared to controls’. Reduction in body weight and BMI following beta-glucan consumption (weighted mean difference [WMD]: −0.77 kg, 95% CI: −1.49, −0.04) and (WMD: −0.62 kg/cm^2^, 95% CI: −1.04, −0.21)
C) Vegetables, fruits, nuts, and seeds (*n* = 11)							
Mytton OT, 2014	(28)	SR and MA of RCTs (*n* = 8, 1,026 participants)	UK	‘To quantify the relationship between changes in vegetable and fruit intake, energy intake and body weight’.	Adults and children	Increase vegetable/fruit consumption, 4–52 weeks, mean 15 weeks. High vegetable and fruit versus low vegetable and fruit.	Mean difference between arms 133 g ‘trials of increased vegetable or fruit consumption, in the absence of guidance to reduce consumption of other foods, result in either a small reduction in body weight or reduced weight gain relative to controls.(mean difference 0.68 kg, 0.15–1.20). No dose response effect’.
Gheflati A, 2019	(29)	SR and MA of RCTs (*n* = 13)	Iran	‘To evaluate the clinical effects of pomegranate consumption on weight and body composition’.	Adults	Pomegranate extract, pomegranate juice, pomegranate vinegar versus placebo capsule, control beverage, water, nothing	‘Participants who consumed pomegranate and its products had no significant reduction in body weight, BMI, WC and body fat percent’.
Onakpoya I, 2017	(30)	SR and MA of RCTs (*n* = 3)	UK	‘To evaluate the evidence for or against the effectiveness of grapefruits (Citrus paradisi) on body weight, blood pressure, lipids’.	Adults with obesity	6–12 weeks fresh grapefruit, frozen juice, and capsules	‘Grapefruit supplementation does not lead to statistically significant reductions in body weight in obese Adults’.
Perna S, 2016	(31)	SR and MA of non-RCTs and RCTs (*n* = 4 and 5, 3 MA) (*n* = 9 studies), 425 participants.	Italy	To review the effects of hazelnut consumption on blood lipid levels as well as on body weight.	Adults	28–84 days with a dosage of hazelnuts ranging from 29 to 69 g/d	‘Out of eight studies with BMI data, six showed absence of difference, and one showed a significant decrease (*P* < 0.05) of 0.5 kg/m^2^, and one showed an increase of BMI and body weight’
Tan SY, 2014	(32)	Narrative review with a number of RCTs assessed	USA	Focuses on the role of nut consumption on appetite, energy intake, energy metabolism, and body weight.	Adults	Nuts and body weight	‘Epidemiologic studies indicate that incorporating nuts into diets on a regular basis does not compromise, and may aid, weight maintenance’. ‘Nut consumption does not promote weight gain’.
Jackson CL, 2014	(33)	Narrative review	USA	‘To determine and reach consensus with regard to nuts’ association with long-term weight change and obesity risk’.	Adults	Nuts and BW change and obesity	‘Based on the available evidence from prospective studies (also supported by RCTs and cross-sectional studies), long-term nut consumption is associated with lower weight gain overweight/obesity’.
Vadivel V, 2012	(34)	Narrative review	Germany	‘To elucidate the link between nut consumption and body weight gain’.	Adults	Nut consumption and body weight gain	‘Several epidemiologic research studies and short-term feeding trials have shown that moderate nut consumption does not increase body weight’.
Kim SJ, 2016	(35)	SR and MA of RCTs (19 reports, 21 trials). Median 6-week follow-up	Canada	To assess studies examining the effects of dietary pulse intake compared with the effects of a comparator diet on body weight.	Adults	Effect of dietary versus isocaloric comparator diets on body weight, WC and BF%	Pooled analysis showed a small weight reduction of 0.34 kg in diets that contained dietary pulses (from 80 to 278 g/d; median intake: 132 g/d or w1 serving/d) compared with diets without a dietary pulse intervention over a median duration of 6 weeks (*P* = 0.03).
Darooghegi Mofrad M, 2019	(36)	SR and MA of RCTs (*n* = 22)	Iran	‘To evaluate the effectiveness of psyllium supplementation on weight loss in adults’.	Adults	Psyllium consumption (whether prescribed through supplements or added to foods) and body weight measures	‘This study did not show a significant effect of psyllium on body weight, body mass index, and waist circumference in comparison with control group in adults’
Mohammadi-Sartang M, 2017	(37)	SR and MA of RCTs (*n* = 45), 3–48 weeks	Iran	‘To help quantify the overall effects of flaxseed products on body composition indices in adults’.	Adults	Flaxseed supplementation whole flaxseed 13–90 g/d, flaxseed oil 1–15.4 g of ALA per day and lignin 50–600 mg/d.	‘Pooled results from the random-effects model showed that BMI was reduced in the flaxseed group compared with the control group (WMD: –0.30 kg/m^2^, 95% CI: –0.53,–0.08, BW 0.99 kg). However, body composition indices were reduced with whole flaxseed consumption only, in trials lasting ≥12 weeks, and if BMI≥ 27’.
Raeisi-Dehkordi H, 2019	(38)	SR (*n* = 25) and MA (*n* = 23) of RCTs	Iran	‘To perform a SR of RCTs that examined the effect of Canola Oil consumption on BW and other anthropometric indexes compared with other sources of dietary fats’.	Adults	Effect of oral ingestion of pure or conventional CO (rapsolje) on BW, both supplements and food?	‘CO intake significantly decreases BW (–0.30 kg); however, CO intake did not significantly affect BMI’.
D) Dairy/dairy proteins (*n* = 9)							
Geng T, 2018	(39)	SR and MA of RCTs (*n* = 37)	Singapore/China/USA	‘To systematically examine the effect of dairy consumption on body weight and body composition. We also sought to compare the effects of trials with and without energy restriction on body composition’.	Adults (*n* = 3,007) 18–79 years old	Dairy consumption and body weight/composition (1–36 months)	This meta-analysis of 37 RCTs with 3,007 adults showed that there was no significant difference in body weight change between the dairy intervention and control groups overall (0.01 kg, 95% CI: −0.25, 0.26). High dairy consumption was associated with decreased body fat (−0.23 kg, 95% CI: −0.48, 0.02) and WC (−1.37 cm, 95% CI: −2.28, −0.46), whereas an increase in lean mass (0.37 kg, 95% CI: 0.11, 0.62). A significant increase in body weight was seen during dairy intervention in 19 RCTs without energy restriction (0.36 kg, 95% CI: 0.01, 0.70 kg, *I*^2^ = 83.1%). In contrast, a significant reduction in body weight that favored dairy products was observed in 16 RCTs that imposed energy restriction (−0.64 kg, 95% CI: −1.05, −0.24 kg, *I*^2^ = 60.2%).
Dewansingh P, 2018	(40)	SR and MA of RCTs (*n* = 36 RCTs and 19 MA)	The Netherlands	To assess the effectiveness of dairy or dairy components on the nutritional status and physical fitness.	Adults 55 years or older	Supplementation with dairy components The two main outcome measurements investigated in this SR were nutritional status including body weight (BW) and body mass index (BMI) and physical fitness, that is, body composition, muscle strength, and physical performance.	‘These systematic review and meta-analysis showed that the dairy components protein and vitamin D have beneficial effects for older adults’. Protein supplementation increased BW by 1.13 kg. This effect was greater after selecting trials. ‘The increase in BW tended to be explained by differences in LBM but only when supplementing doses of protein higher than 20 g/d’.
Stonehouse W, 2016	(41)	SR and MA of RCTs (*n* = 27 Q and *n* = 24 MA). Median 16 weeks Total *n* = 1,278, BW changes, *n* = 864)	Australia	To investigate the effects of dairy food/supplements during energy restriction on body weight and composition in 18–50-year-old.	Adults 18–50 years Mainly women (80%)	Calorie restriction (most > 500 kcal/d), dairy foods (*n* = 21), and dairy supplements (*n* = 6) and BW	‘Overall (when dairy food and supplement studies were pooled), increased dairy intake combined with energy restriction resulted in a significantly greater reduction in body weight (Figure 2) and body fat mass (Figure 3) compared to control interventions (body weight: –0.92 kg [–1.63, –0.20 kg], smaller loss of lean mass (0.36 kg, 0.01–0.71)’
Booth AO, 2015	(42)	SR and MA of RCTs (*n* = 41)	Australia	‘This review comprehensively and systematically analyses the available evidence assessing the effects of dairy and added Ca supplementation on body weight’.	Adults	Dairy, added calcium, and body weight	‘In summary, a robust meta-analysis method with large subject numbers showed that there was no evidence that increased Ca provision in the form of supplements or dairy foods reduces body weight or body fat. There was some evidence that consumption of approximately 3 servings/d of reduced fat dairy foods (approximately 1300 mg/d Ca) in the presence of energy restriction resulted in a small, but significant greater loss of body fat mass over a short period of 4 months. This indicates that low fat dairy foods can be included as part of a healthy weight loss diet without having negative effect on body weight or body composition in the short term’.
Chen M, 2012	(43)	MA of RCTs (*n* = 29) *N* = 2,441 participants	USA	‘To evaluate whether increasing the consumption of dairy products could promote weight loss’.	Adults	Effect dairy consumption on body weight and body fat in adults	‘This meta-analysis does not support the beneficial effect of increasing dairy consumption on body weight and fat loss in long-term studies (≥1 year) or studies without energy restriction’. ‘There was no significant difference in body weight changes between the dairy intervention and control groups (–0.14 kg; 95% CI: –0.66, 0.38 kg; Figure 2A); however, a significant reduction that favored dairy products in body fat was shown (–0.45 kg; 95% CI: –0.79, –0.11 kg; Figure 2B)’.
Abargouei AS, 2012	(44)	SR and MA (*n* = 14) of RCTs *N* = 883	Iran	To summarize the evidence on the effect of dairy consumption on body weight and composition and to identify possible sources of heterogeneity between studies.	Adults, 18–85 years	Effect of dairy consumption on body weight and composition	‘In conclusion, our systematic review and meta-analysis on RCTs indicated that increasing dairy consumption without energy restriction might not lead to a significant change in weight and body composition, whereas inclusion of dairy products in weight loss energy-restricted diets would result in a greater reduction of weight, fat mass and WC and gain in lean body mass compared with the conventional weight loss diets’. ‘Slightly greater weight loss among those with high dairy intake compared with those with low dairy intake (–0.61 kg (95% confidence interval (CI): –1.29, 0.07)’.
Bendtsen LQ, 2013	(45)	Review	Denmark	‘To examine the existing evidence from controlled clinical trials investigating the effects of consumption of dairy protein (total dairy protein, whey, and/or casein) and other protein sources on …., body weight, and body composition’.	Not addressed	Effect of dairy consumption on body weight and composition and more	Despite good evidence to support that protein is beneficial in increasing and maintaining weight loss due to effects on appetite regulation and energy expenditure, data are inconclusive with regard to the effects of various protein types.
Dougkas A, 2011	(46)	SR and MA of studies with various study designs (cross-sectional, prospective observational), *n* = 18 and intervention trials	UK	‘The present review specifically examines the evidence from epidemiological studies and intervention trials that have investigated the relationship between dairy product consumption and dietary Ca, and measures of adiposity’.	Adults	Dairy product consumption and dietary Ca, and measures of adiposity.	‘Although inconsistencies between studies certainly exist, the overall assessment of the epidemiological evidence is suggestive of a modest negative association between dairy consumption and body weight. The overall linear regression analysis, based on the 18 trials that examined dietary Ca (with the majority of dietary Ca derived from dairy products), indicates that an increase in Ca intake from 400 to 1,200 mg/d would be associated with a decrease in BMI from 25.6 to 24.7 kg/m^2^. Evidence derived from intervention studies without energy restriction does not predict any effect of dairy products on either weight loss or weight gain. During energy restriction, although the results are still inconsistent, there are indications of a possible beneficial effect of dairy products in weight loss treatments whilst maintaining lean tissue in an overweight population’.
Anderson GH, 2011	(47)	Narrative review (Nestle)	Canada	‘Review of the role of dairy in the regulation of body weight and the role of cow’s milk proteins in the regulation of satiety, food intake, blood glucose and their mechanisms of action’.	Adults	Dairy and body weight	‘Several epidemiological studies of adults have reported an inverse in association between frequent dairy intake and adiposity as measured by the body mass index’.
E) Diets according to food or macronutrient composition (low GI-diets, vegan/vegetarian, low-carb, and low-fat) (*n* = 10)							
Vega-López, S, 2018	(48)	Review of RCTs and observational studies comparing foods, meals, or diets with distinct GI (*n* = 73) 2006–2018 (BW, *n* = 8)	USA	‘To summarize the most recent evidence for short-term and long-term (e.g. weight) health effects associated with different types of GI diets’.	Adults	Different GI diets Weight loss 8-week to 18-month	‘Findings regarding an association between GI or GL and body weight are equivocal (observational studies). Although one shorter-term study (8-week) suggested a benefit from lowering the GI of the diet for greater weight loss, highly-controlled feeding interventions (3–18 months) suggested that manipulating the GI does not make a difference in weight-related outcomes (Table 4)’.
Chiavaroli L, 2018	(49)	SR and MA of RCTs (*n* = 32, 2,448 participants, *n* = 9 negative energy balance) median follow-up of 12 weeks (IQR 9–21)	Canada	‘To quantify the effect of pasta alone or in the context of low-GI dietary patterns on body weight and measures of adiposity relevant to the prevention and management of overweight and obesity’.	Adults	Pasta alone (none) or in the context of low-GI dietary patterns Primary outcome was body weight	Pasta in the context of low-GI dietary patterns reduced body weight by −0.63 kg (−0.84 to –0.42 kg; *P* < 0.001) compared with higher-GI control. 24 trials with <24 weeks’ follow-up weight reduction similar to eight trials with ≥24 weeks’ follow-up (−0.63 kg vs. −0.57 kg, respectively).
Munsters MJ, 2014	(50)	Narrative review	Netherlands	1. Discuss the concepts of body weight regulation, substrate partitioning,2. Address dietary strategies to …. and to achieve and maintain a healthy body weight.	General population	Dietary strategies that may improve metabolic profile and BW in obesity, and to achieve and maintain a healthy body weight.	‘A growing body of evidence suggests that dietary strategies with the aim to reduce postprandial insulin response and increase fat oxidation, and that tend to restore metabolic flexibility, have a place in the prevention and treatment of obesity and associated metabolic disorders’.
Barnard ND, 2015	(51)	SR and MA of clinical trials (*n* = 4), rheumatoid arthritis (*n* = 2), people with overweight or diabetes (*n* = 2)	USA	‘To identify the body of data from clinical trials using vegetarian (including vegan) diets as interventions and to quantify the weight loss resulting from the prescription of these diets in adults, independent of the confounding effects of exercise or caloric limits’.	Adults	Vegan/vegetarian diets of 3–13 months duration without energy intake limitations versus untreated controls (2 low-fat vegan, 1 raw vegan, 1 vegan/lacto-vegetarian)	‘Our meta-analysis showed that the prescription of vegetarian or vegan diets was associated with a mean weight reduction of 3.4 (2.4–4.4) kg in an intention-to-treat analysis and 4.6 (3.8–5.4) kg in a completer analysis’.
Kirkpatrick CF, 2020	(52)	Review of MAs of RCTs, *n* = 17 (*n* = 8 without diabetes or prediabetes)	USA	‘Review evidence on the effects of CHO-restricted dietary patterns compared to dietary patterns with higher CHO content on body weight and glycemic control’.	Adults with overweight/obesity with/without diabetes/prediabetes	CHO-restriction versus HCLF very-low-CHO <25–50 g CHO/d; low CHO 50–130 g CHO/d)	‘CHO-restricted interventions may result in greater weight loss and glycemic control in the short term (≤6 months) compared to HCLF interventions. However, in the long term (especially those > 12 months), evidence does not support the view that CHO-restricted dietary patterns are superior to HCLF dietary patterns for weight loss or T2D management’.
Kirkpatrick CF, 2019	(53)	Review of MA of RCTs and non-RCTs, *n* = 14 (*n* = 6 without diabetes/prediabetes)	USA	Review the characteristics of low- and very-low-CHO diets and their impacts on metabolic pathways and on weight loss.	Adults with overweight/obesity	Low-CHO/high-fat versus high-CHO and/or low fat (mean CHO intake in the low- and very-low-CHO diet groups at the end of follow-up exceeded 50 g/d in all except one study)	‘Short-term (≤6 months) hypocaloric low-CHO and very low-CHO diets may result in greater weight loss than hypocaloric high-CHO, low-fat (HCLF) diets. Longer-term (>6 months) results suggest that low-CHO and very-low-CHO diets may result in weight loss that is equivalent to that of HCLF diets. Very-low-CHO diets are difficult to maintain and are not clearly superior for weight loss compared with diets that allow a higher amount of CHO in adults with overweight and obesity with or without diabetes. Moreover, three separate observational studies, including a large prospective cohort study with long-term follow-up, have shown that a very-low-CHO intake is associated with increased all-cause mortality’.
Mansoor N, 2015	(54)	MA of RCTs (*n* = 11, 1,369 participants)	Norway	‘To compare a typical LC diet defined as a CHO intake of 20–30 g/d in the first phase or <20% of total energy with traditional LF diets composed of <30% of energy as fat and limited energy content, as well as determine the effects on long-term weight loss and several CVD risk factors in healthy adults’.	Adults	Low-Carb versus Low_Fat, duration 6–24 months	‘Compared with subjects on LF diets, subjects on LC diets experienced significantly greater weight loss after 6 months to 2 years of intervention’ (WMD –2 17 kg; 95% CI –3·36, –0·99)’
Martens EA, 2014	(55)	Narrative review	Netherlands	‘To specify how protein diets can be applied as a clinical approach for body weight loss and weight maintenance’.	Not addressed	Protein diets and body weight	‘In adults, a protein intake of 0.8–1.2 g/kg/d is sufficient to sustain satiety, energy expenditure, and FFM, independent of a dietary ‘low-carb’ content. This implies that protein intake does not need to be exceptionally high to be used for body weight management’.
Krishnan S, 2014	(56)	Narrative review	USA	‘We chose studies that fed high-fat diets and reported fat oxidation, EE, and weight maintenance’.	Not addressed	High-fat diets and weight maintenance	In conclusion, a high MUFA or PUFA diet appears to be more metabolically beneficial compared to a high SFA diet in terms of EE and weight maintenance.
Kim JE, 2016	(57)	SR and MA of RCTs (*n* = 20)	USA	‘To assess and evaluate the effects of protein intake on dietary energy restriction–induced changes in body mass, lean mass, and fat mass in groups of adults with a mean age 50 years and older’.	Adults	Protein intake modified by protein supplementation and/or a prescribed higher protein diet; average energy deficit 500–750 kcal/d. The length of the energy restriction interventions ranged from 8 weeks to 2 years.	‘Results from this systematic review and meta-analysis of findings from RCTs consistently indicate that older men and women better retain lean mass while losing body mass during periods of diet-induced energy restriction when they consume higher protein versus normal protein diets’.
F) Macronutrient composition – fats (*n* = 4)							
Hooper L, 2015	220 (58)	SR and MA of RCTs (*n* = 32) and cohort studies (*n* = 25)	UK	‘To assess the effects of proportion of energy intake from fat on measures of weight and body fatness (including obesity, WC, and BMI in people not aiming to lose weight, using all appropriate randomized controlled trials (RCTs) and cohort studies in adults, children and young people’.	Children and adults	Proportion of energy intake from fat on body weight	‘There is consistent evidence from RCTs in adults of a small weight-reducing effect of eating a smaller proportion of energy from fat; this was seen in almost all included studies and was highly resistant to sensitivity analyses. The effect of eating less fat (compared with usual diet) is a mean weight reduction of 1.5 kg (95% CI –2.0 to –1.1 kg), but greater weight loss results from greater fat reductions. The size of the effect on weight does not alter over time and is mirrored by reductions in body mass index (BMI) (–0.5 kg/m^2^, 95% CI –0.7 to –0.3) and waist circumference (–0.3 cm, 95% CI –0.6 to –0.02). Included cohort studies in children and adults most often do not suggest any relationship between total fat intake and later measures of weight, body fatness or change in body fatness. However, there was a suggestion that lower fat intake was associated with smaller increases in weight in middle-aged but not elderly adults, and in change in BMI in the highest validity child cohort’.
Hooper L, 2012	(59)	SR and MA of RCTs (*n* = 34, 1 in children) and CHS (*n* = 13, 3 in children)	UK	SR was needed of all available evidence of longer-term effects of total fat intake on body fatness, in studies not intending that participants lose weight.	Children and adults	Total fat intake and body fatness	‘Diets lower in total fat on average reduced body weight by 1.6 kg, BMI by −0.51, and waist circumference by 0.3 cm. These effects were from randomised controlled trials in which weight loss was not an intended outcome, suggesting that they occur in people eating normal diets and the direction of effect on weight was consistent regardless of subgroups or sensitivity analyses’.
Martınez-Victoria E, 2012	(60)	SR of RCTs (*n* = 5 body weight)	Spain	‘To determine the effect of n-3 PUFA supplementation or diets enriched in n-3 PUFA on body weight in adults, and…’	Adults	n-3 PUFA and body weight	‘The results of …. do not provide us with data robust enough as to conclude that n-3 PUFA can modify, and particularly reduce, body weight. Marked differences in experimental design, intervention type and duration, baseline characteristics of the participants (degree of obesity, associated condition, etc.), attrition rate, dose of n-3 PUFA and EPA/DHA ratio, make the results inconclusive and, in some cases, discordant’.
Mumme K, 2015	(61)	SR and MA of RCTs (*n* = 13)	New Zealand (?)	‘To conduct a SR and meta-analysis of randomized controlled trials comparing the effects of MCTs, specifically C8:0 and C10:0, to long-chain triglycerides (LCTs) on weight loss and body composition in adults’.	Adults	MCTs, specifically C8:0 and C10:0, versus LCTs on weight loss 4–16 week	Consuming MCTs as part of a diet compared with LCTs may result in a small average reduction in body weight of 0.51 kg (range 0.80–0.23 kg) over an average 10-week period.
G) Water, tea, coffee, and alcohol (*n* = 5)							
Muckelbauer R, 2014	(62)	SR of longitudinal (*n* = 4) and cross-sectional (*n* = 9) Q synthesis	Germany	‘To summarize the evidence from existing studies on the association between the consumption of water as a beverage and body weight outcomes’	Children and adolescents	Water and body weight	‘The longitudinal association seemed to be inverse, indicating that increased water consumption might reduce the risk for excessive weight gain in childhood. However, evidence was very limited because this trend was shown by only three longitudinal studies, while one longitudinal study did not find this association. Overall, the evidence for any causal association between water consumption and body weight outcomes is very low due to the sparse existing literature on this association and due to the type of existing studies being mainly cross-sectional’
Muckelbauer R, 2013	(63)	SR of RCTs (*n* = 3), non-RCT (*n* = 1), observational cohort (*n* = 1), CSCS (*n* = 6), reviews (*n* = 2)	Germany	‘To systematically summarize all existing evidence of the association between dietary water consumption and weight-related outcomes in adults’.	Adults	Water and body weight	‘Of the studies with populations participating in a program for weight loss or maintenance, 2 studies – 1 nonrandomized interventional trial and 1 observational longitudinal study – showed that increased water consumption has the potential to reduce body weight’.
Yang CS, 2016	(64)	Narrative review	USA/China	‘This article reviews the evidence and discusses the molecular mechanisms for the mitigation of overweight and MetS, as well as related prevention of diabetes and CVDs by different types of tea’.	Not defined	Tea and body weight referred	‘Tea consumption at the levels of three to four cups (600–900 mg tea catechins) or more a day has shown to reduce body weight gain, alleviate MetS, and reduce the risk for diabetes and CVDs’.
Hursel R, 2013	(65)	Narrative review	The Netherlands	‘Results from different types of studies, such as intervention studies and observational studies, are discussed to give a detailed overview of the evidence on tea as weight-controlling ingredient’.	Adults	Tea and body weight	‘Studies and meta-analyses of catechin- and caffeine-rich teas (CCRTs) in most cases showed improved anthropometric variables such as BW, BMI, body fat mass, and waist:hip ratio’.
Sayon-Orea C, 2011	(66)	SR of 14 cross-sectional studies, 13 prospective cohort studies, and four intervention trials	Spain	‘To systematically review the effects of alcohol consumption on body weight’.	Adults	Alcohol and body weight	‘Most of the cross-sectional studies found a positive association between alcohol consumption and body weight or measures of abdominal adiposity (WC or WHR), especially in heavy drinkers and binge drinkers, whereas moderate consumption was either negatively associated or not associated with body weight or abdominal adiposity’.
H) Meat							
An R, 2020	(67)	SR of RCTs (*n* = 7) and observational studies (*n* = 5), MA (*n* = 8)	USA	‘Aimed at systematic identification and synthesis of the scientific evidence on pork consumption in relation to body weight’	Adults aged 18 years and older	Pork consumption/body weight and composition	‘Meta-analysis found that among the experimental studies without energy restrictions, pork intake was associated with a reduction in body weight by 0.86 kg and body fat percentage by 0.77%; among the experimental studies with energy restrictions, pork intake was associated with a reduction in body weight by 5.56 kg, lean mass by 1.50 kg, and fat mass by 6.60 kg; and among the observational studies, pork intake was not associated with overweight or obesity status’.

An overall summary of the results within each category of intervention/exposure is shown in Table 1, and the main findings are presented below.

A) Sugar intake, SSBs, and NNSs

#### Sugar and sugar sweetened beverages

Two SRs and MAs of the effects of fructose (12) and dietary sugars (13), respectively, and one SR of reviews (10) showed evidence that increased intake of sugar or SSBs in short-term clinical trials was associated with modestly increased body weight (0.8 kg) (13), and that reducing sugar intake was associated with modestly reduced body weight (0.8 kg) (13). Importantly, these results seemed to be explained by changes in energy intake rather than an independent effect of monosaccharides or disaccharides on body weight (8, 12, 13).

#### Non-nutritive sweeteners

One recent SR and MA of 20 randomised controlled trial (RCTs) (15) including 2,914 participants with the duration of 4–77 weeks showed that participants consuming NNS showed small but significant weight/body mass index (BMI) differences favoring NNS compared with non-users (–1.3 kg). However, NNS versus placebo/no intervention and NNS versus water produced no effect. When comparing NNS with sucrose, significant weight/BMI differences appeared favoring NNS. The effect was more evident when NNS was used as a substitute for sucrose, especially in adults, in subjects presenting with overweight/obesity, and in those following an unrestricted diet (≈ 1 kg). Two other SRs and MAs of RCTs and prospective cohort studies (18, 19) showed similar results in RCTs, with no convincing evidence that low-calorie sweeteners were associated with weight gain in prospective cohort studies.

B) Whole grain and fiber

A recent MA of RCTs and qualitative analysis of prospective cohort studies (20) reported no significant effect of short-term whole grain intervention on BMI-change in adult people. By contrast, higher whole grain intake was significantly inversely associated with BMI in prospective cohort studies.

Regarding fiber, pooled analysis of 62 RCTs (*n* = 3,877) showed small reductions in body weight (−0.33 kg), BMI (−0.28), and waist circumference (−0.63 cm), at a median dose of 6.7 g viscous fiber/d (range: 0.8–36 g/d) for a median (range) duration of 10 (4–52) weeks (25). Foods with high fiber content are discussed in the next paragraph (C).

C) Vegetables, fruits, nuts, and seeds

*Vegetables and fruits*. Only one MA of eight RCTs (mean follow-up 15-week, up to 1-year) compared high versus low intake of vegetables and fruits, mean difference 133 g (28), showing a small reduction in body weight or reduced weight regain (0.7 kg, 95% CI 0.2–1.2).

*Concerning nuts*, three narrative reviews (32–34) discussed the role of nut consumption on body weight. Based on the available evidence from prospective studies, long-term nut consumption is associated with lower weight gain (33). Bottom line data from epidemiologic studies suggest that a diet including nuts does not increase and could potentially support a stable body weight. However, long-term RCT studies are needed to evaluate the effects of daily realistic consumption of nuts on body weight as a primary outcome (32).

*Seeds*. As shown in a SR and MA of 28 of 45 RCTs, the consumption of flaxseed may affect body weight and body composition (37). A modest effect was found when supplementing with either whole flaxseed or flaxseed oil, minus 1.0 (–1.7 to –0.3) kg. Subgroup analyses showed that flaxseed consumption reduced body weight, BMI, and waist circumference (WC) only among participants with BMI ≥ 27 kg/m^2^, and longer interventions showed larger effects of whole flaxseed in the diet (37). By contrast, for psyllium, no effect on body weight was observed (36).

D) Dairy/dairy proteins

Dairy consumption was suggested to have a weak inverse association with body weight gain in a review of studies of different study design (46). Furthermore, increased weight loss and preservation of lean body mass during energy restriction were reported with increasing intake of dairy products. However, a more recent MA of 37 RCTs including 3,007 adults showed that overall, there was no significant difference in body weight change between the dairy intervention and control groups (0.01 kg, 95% CI: −0.25, 0.26), but high dairy consumption was associated with an increase in lean mass (0.37 kg, 95% CI: 0.11, 0.62) (39). By contrast, during energy restriction of ≤600 kcal in 16 RCTs, dairy included in the diet resulted in reduction in body weight, −0.64 kg, 95% CI: −1.05, −0.24 kg (39). Another MA of RCTs with energy restriction reported 0.9 kg larger weight loss and smaller loss of lean mass (0.4 kg) in the dairy food and supplements groups as compared with energy restricted controls (41).

E) Diets according to food or macronutrient composition

*Glycaemic index/load*. Findings regarding any association between Glycemic Index (GI) and Glycemic Load (GL) and body weight are equivocal (observational studies). Although one shorter-term study (8-week) suggested a benefit from lowering the GI of the diet for greater weight loss, longer-term highly controlled feeding interventions suggested that manipulating the GI does not make a difference in weight-related outcomes (48). By contrast, SR and MA of 32 RCTs median follow-up of 12 weeks (Interquartile range (IQR) 9–21 weeks) demonstrated that pasta in the context of low-GI dietary patterns slightly reduced body weight, −0.63 kg (−0.84 to –0.42 kg) compared with higher-GI controls (49).

*Vegan/vegetarian*. A(n) MA of four clinical trials, two including patients with rheumatoid arthritis and two including employees of the Government Employees Insurance Company (GEICO) with overweight or type 2 diabetes, showed relatively large reductions in body weight after 3–13 months treatment with vegan and/or vegetarian diets compared with controls, –3.4 (–4.4 to –2.4) kg (51, 68–71). The two studies of people with overweight or type 2 diabetes prescribed a low-fat vegan diet for 18 or 22 weeks in the intervention groups, resulting in 2.8 and 5.3 kg larger mean weight loss than in the control groups (68, 69).

*Low-carb-high fat versus high-carb-low fat.* A recent review of MAs of RCTs (*n* = 17) (*n* = 8 without diabetes or prediabetes) concluded that intervention with low-carb diets resulted in more reduction in body weight in the first 6 months compared with high-carb-low-fat diets (HCLF) (52). However, in interventions with longer duration, most pronounced in studies >12 months, low-carb diets did not have better effect on weight reduction compared with HCLF diets. This is partly in contrast to the results of a Norwegian MA of 11 RCTs, concluding that typical low-carb diet interventions (starting with CHO ≤20–40 g/d in the first phase or <20% of total energy intake) lasting 6–24 months resulted in a larger mean reduction in body weight compared with typically low-fat diets (<30% of energy as fat), –2.17 (95% CI –3.36 to –0.99) kg (54).

F) Macronutrient composition – fats

*Fats.* The most recent SR and MA of RCTs and cohort studies regarding the effects of proportion of energy intake from fat on measures of weight and body fatness showed a moderate effect of reducing the proportion of dietary fat, –1.5 kg (95% CI –2.0 to –1.1 kg) in comparison with the usual diet (58). A dose-response effect was observed with larger reductions in body weight as a result of lower proportions of fat.

G) Water, tea, coffee, and alcohol

Few reviews in the search addressed the potential association among the intake of water (62, 63), tea (64, 65), or alcohol (66). Two studies with populations participating in a program for weight loss or maintenance, one non-randomized interventional trial, and one observational longitudinal study suggested that increased water consumption may have the potential to reduce body weight (63). Regarding the potential associations among tea, coffee, alcohol, and BW, the evidence is sparse or absent.

H) Meat

One SR of seven experimental and five observational studies and MA of eight (six experimental) studies (67) showed null or inverse associations between pork intake and body weight, body fat percentage, and fat mass. In the experimental studies with energy restrictions, pork intake was associated with reduced body weight by 5.6 kg (95% CI 0.6–10.6), lean mass by 1.5 kg (95% CI 1.4–1.6), and fat mass by 6.6 kg (95% CI 6.4–6.8). Pork intake was not associated with overweight/obesity in the observational studies.

## Discussion

### Main findings related to research questions

First, the overall body of evidence based on findings from SRs and MAs of observational and clinical intervention studies indicates that changes in intakes of specific nutrients (sugar, fiber, protein, and fat) and/or foods (SSB, fiber rich food, and vegetables) are associated with modest or small short-term changes in body weight in the general population (with or without obesity/overweight), while long-term studies are generally lacking. Second, no study in our search assessed the association between body weight (exposure) and intakes of specific nutrients or foods (outcomes). Third, limited evidence suggests, but does not prove, that some foods or nutrients may have specific effects on body weight or body weight measures independent of caloric content (e.g. nuts and dairy).

### Discussion of specific findings

#### Nuts

Nuts alone or as part of dietary patterns have been recommended for cardiovascular health, but it has been questioned whether their high energy density may contribute to weight gain. By contrast, two narrative reviews suggested that nut consumption does not promote weight gain (32), and that long-term nut consumption is associated with reduced weight gain (33). A recent SR, MA, and dose-response meta-regression of seven prospective cohorts and 86 RCTs (not covered by our search) concluded that nuts were associated with slightly lower incidence of overweight/obesity (RR 0.93 [95% CI 0.88 to 0.98], *P* < 0.001) in prospective cohorts (72). However, MA of RCTs did not show any significant effect of nuts on body weight. Meta-regression showed that higher nut intake was significantly associated with reductions in body weight and body fat (72). The lack of association between nut consumption and overweight/obesity may have several explanations. First, nuts are rich in protein and fiber, both associated with increased satiety. Second, proteins have a high diet-induced thermic effect. Third, the physical structure of nuts may contribute to fat malabsorption. In view of these, the calories obtained by the body from nut consumption may be overestimated by approximately 16–25% depending on the nut type and form (72).

### Dietary patterns

#### Vegan/vegetarian diets

The MA including four controlled intervention studies assessing vegan and vegetarian diets requires attention since –3.4 kg is the largest effect presented in this ScR (51). However, these findings might not be generalizable to the general population. First, two of four studies in the MA included <30 patients with rheumatoid arthritis who were allocated to restricted vegan diets (70, 71). Second, the two other studies assessed the effect of intensive workplace interventions in 113 and 291 participants with overweight and/or type 2 diabetes (68, 69). A recent SR by Tran et al. (73) (not covered by our search) included these four studies in addition to 15 other clinical trials assessing the effects of plant-based diets on weight status. The majority of these were RCTs comparing a low-fat vegan diet with an omnivore diet in participants with overweight, type 2 diabetes, and/or cardiovascular disease. The authors concluded that a shift to a plant-based diet may support a healthy body weight but did not perform an MA (no effect size was given). Factors that may improve weight status are higher intakes of fiber, unsaturated fats, and plant proteins, together with increased thermic effect of foods, and reduced intake of energy, saturated fats, and animal proteins (73). These beneficial effects may be partly mediated by a less obesogenic gut-microbiota composition (73).

#### The Mediterranean and Nordic diets (not covered by our search)

A recent SR of eight intervention studies (8-week to 3-year follow-up) and 47 observational studies, the majority of low quality, concluded that ‘there is only limited evidence of a beneficial effect of following a traditional Mediterranean diet to maintain a healthy body weight in childhood’ (74). Furthermore, an SR of five RCTs in adults concluded that the Mediterranean diet resulted in similar weight loss as comparator diets in individuals with overweight or obesity trying to lose weight (75).

The healthy Nordic diet was described in 2010 to include typical foods in the Nordic countries such as fruits and berries, vegetables, legumes, low-fat dairy products, and fatty fish (76). Furthermore, the Nordic diet included oats, barley, almonds, and psyllium seeds (76). SR and MA of five RCTs (42–182 days follow-up) showed that those adhered to the Nordic diet lost 1.8 kg [95% CI – 2.9, –0.7, *P* = 0.001] more weight compared with controls on a typical or average Danish diet (77).

In the current ScR, small reducing effects on body weight were found for vegetables or fruit (28), whole grains (20), and viscous fibers (25). However, it cannot be excluded that a dietary pattern including these factors together could result in a combined beneficial effect on body weight. The impact of similar dietary patterns on satiation, satiety, and digestibility (26, 78) may explain these effects, and they all affect energy intake or access of energy in different ways (79, 80).

### Conclusions in context of NNR2012

First, one recent MA of RCTs partly confirmed the NNR2012, arguing that ‘the evidence linking a higher dietary fibre intake to reduced weight gain is clear’ (prospective studies), but, notably, the average effect size was small (e.g. 0.33 kg) after a median of 10 weeks and a median dose of 6.7 g viscous fiber a day (25). Second, in line with the NNR2012, our updated search did not find any significant associations between macronutrient composition and long-term weight change. Third, in agreement with NNR2012, results from intervention studies not designed for intentional weight loss show that reduced total fat intake was associated with a modest (1.5 kg) weight reduction (58). Fourth, in agreement with NNR2012, reduced intake of sugar and SSBs was associated with modest weight loss, which was explained by reduced calorie intake. Fifth, our results partly confirmed that fiber-rich foods such as fruit and vegetables are associated with small reductions in body weight or weight gain (28). Sixth, our updated search confirmed that sugar-rich foods and drinks are associated with increased weight gain in the long term. However, with the exception of one study assessing the effect of pork (67), no studies were found including red meat and processed meat. This review was limited by high heterogeneity and a low number of publications, but it found that pork intake was associated with a reduction in body weight by approximately 1 kg, and not associated with overweight or obesity (67).
